# Intact Excision of a Mesenteric Pseudocyst

**DOI:** 10.7759/cureus.40615

**Published:** 2023-06-19

**Authors:** Kumar Kaushik, Arvind Pratap, Bitan Naik, Anumanchi Datta Sai Subramanyam, Mumtaz A Ansari

**Affiliations:** 1 General Surgery, Institute of Medical Sciences, Banaras Hindu University (BHU), Varanasi, IND; 2 Pathology, Institute of Medical Sciences, Banaras Hindu University (BHU), Varanasi, IND

**Keywords:** intact excision, abdominal cyst, mesentery, laparotomy, excisional biopsy, mesenteric pseudocyst

## Abstract

Mesenteric cysts are detected in all age groups with almost equal incidence in both genders. Although a rare abdominal growth, it is commonly found in the fifth to seventh decades of life. These are mostly small (asymptomatic) with a 3% chance of malignant transformation. With the increase in the size of the cyst, nonspecific complaints of abdominal pain, distention, discomfort, nausea, vomiting, flatulence, constipation, or diarrhea may develop. Owing to the varied presentation and lack of pathognomonic clinical, laboratory, or imaging findings, these are difficult to diagnose. The subtype mesenteric pseudocyst is even rarer with a reported incidence of less than 1 out of 250,000 hospital admissions and can be found anywhere along the mesentery from the duodenum to the rectum. Etiology is either traumatic or infectious. Incidental diagnosis during abdominal imaging or laparotomy is common. However, it warrants immediate surgical intervention when infected or ruptured. Complete excision of the cyst is the treatment of choice. Here, we report an interesting case of a middle-aged gentleman who had been repeatedly evaluated for a tense abdomen with exudative ascites. Following decompression, he presented to us with a large obliquely mobile mass in the abdomen. The diagnosis was made by clinical and radiological findings and confirmed by histopathological examination of the intact, excised specimen post-laparotomy.

## Introduction

Mesenteric pseudocysts are rare abdominal tumors that can arise from mesenteric tissues. They are typically benign, fluid-filled cysts that can cause abdominal pain, nausea, vomiting, and other nonspecific abdominal symptoms [1]. The diagnosis of mesenteric pseudocysts can be challenging, as they can mimic other abdominal masses and present with nonspecific symptoms.

Surgical excision is the preferred treatment for mesenteric pseudocysts, but the optimal approach to surgery remains controversial. There are various techniques for cyst excision, including partial or complete cystectomy, marsupialization, and simple drainage. Laparoscopic methods are recently better accepted than laparotomy [2]. However, intact excision of the cyst is a preferred approach that involves en-bloc or complete resection of the entire cyst without spillage [3].

Although the pseudocyst by definition lacks a true wall, which makes intact excision practically impossible, we report a case of mesenteric pseudocyst where intact removal could be achieved.

## Case presentation

A 52-year-old gentleman, a resident of Gorakhpur, Uttar Pradesh, presented to the surgery outpatient department with a complaint of low-grade intermittent fever and a gradually increasing abdominal distention for the past nine months. The fever was insidious in onset and relieved after taking oral medications. The patient also reported a cough for two weeks initially, nine months ago. He had been receiving empirical treatment for abdominal tuberculosis for the past nine months and was on anti-tubercular therapy (ATT).

**Figure 1:**
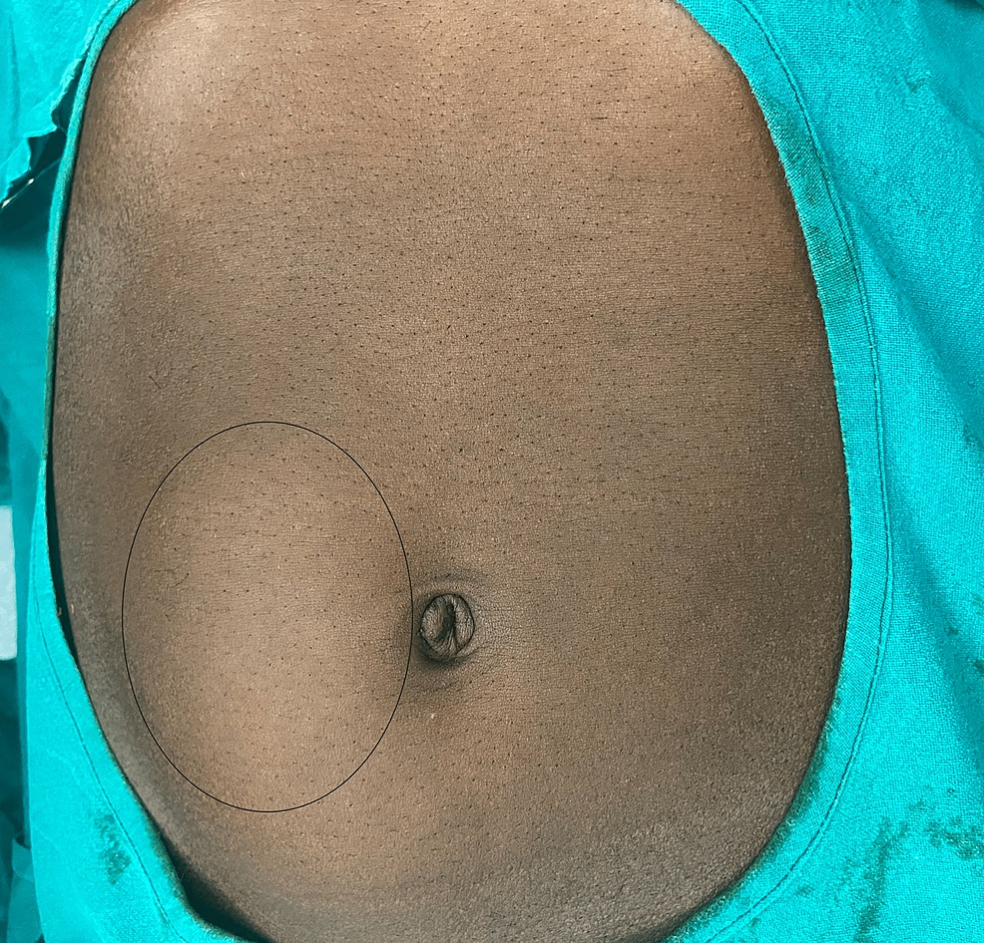
Solid line encircles abdominal mass in the umbilical, right lumbar, and right iliac fossa region

On examination, his general physical examination was unremarkable. The abdomen was distended, and a 15x12 cm mass was palpable in the umbilical, right lumbar, and iliac fossa region (Figure 1).

The mass was non-tender, firm, and intra-peritoneal. It had a smooth surface and ill-defined margins. It was obliquely mobile with restricted mobility along the line of mesenteric attachment. No hernias were evident, and bowel sounds were normal. The digital rectal examination did not reveal any abnormality.

Based on the patient's clinical history and examination findings, four differential diagnoses were considered, namely, peritoneal tuberculosis, mesenteric cyst, gastrointestinal stromal tumor (GIST), and peritoneal hydatid cyst.

**Figure 2:**
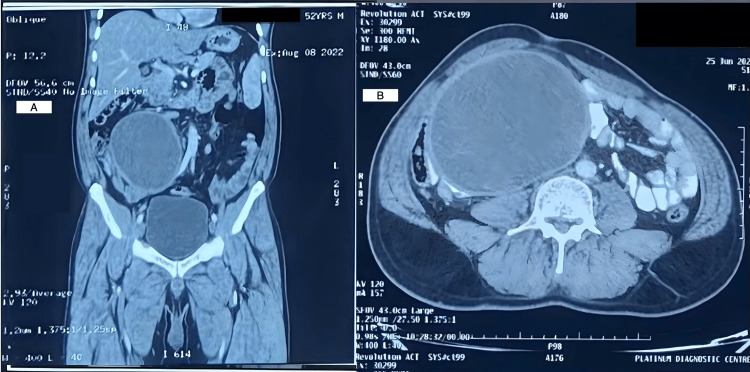
Post-contrast CT images in A) coronal and B) axial section CECT shows a large (11.5 x 13.0 x 15.5cm), well-defined, well-marginated, encapsulated hypodense lesion showing enhancement in the thickened walls of the lesion in post-contrast images. It shows no obvious septations, calcifications, solid components, and no surrounding infiltration. CECT: contrast-enhanced computed tomography

The routine blood tests were normal. The imaging studies, including contrast-enhanced computed tomography (CECT) whole abdomen (Figure 2), showed a large, well-defined, encapsulated hypodense lesion in the right lumbar and iliac fossa region, which was likely an infected mesenteric cyst or hydatid cyst. The liver was enlarged in size with a diffuse decrease in tissue attenuation, suggestive of fatty hepatomegaly. The abdominal ultrasonographic review did not reveal any additional findings. The Echinococcus immunoglobulin G (IgG) was positive. Hence, we considered peritoneal hydatid cysts (type 1) and mesenteric cysts as probable diagnoses [4].

**Figure 3:**
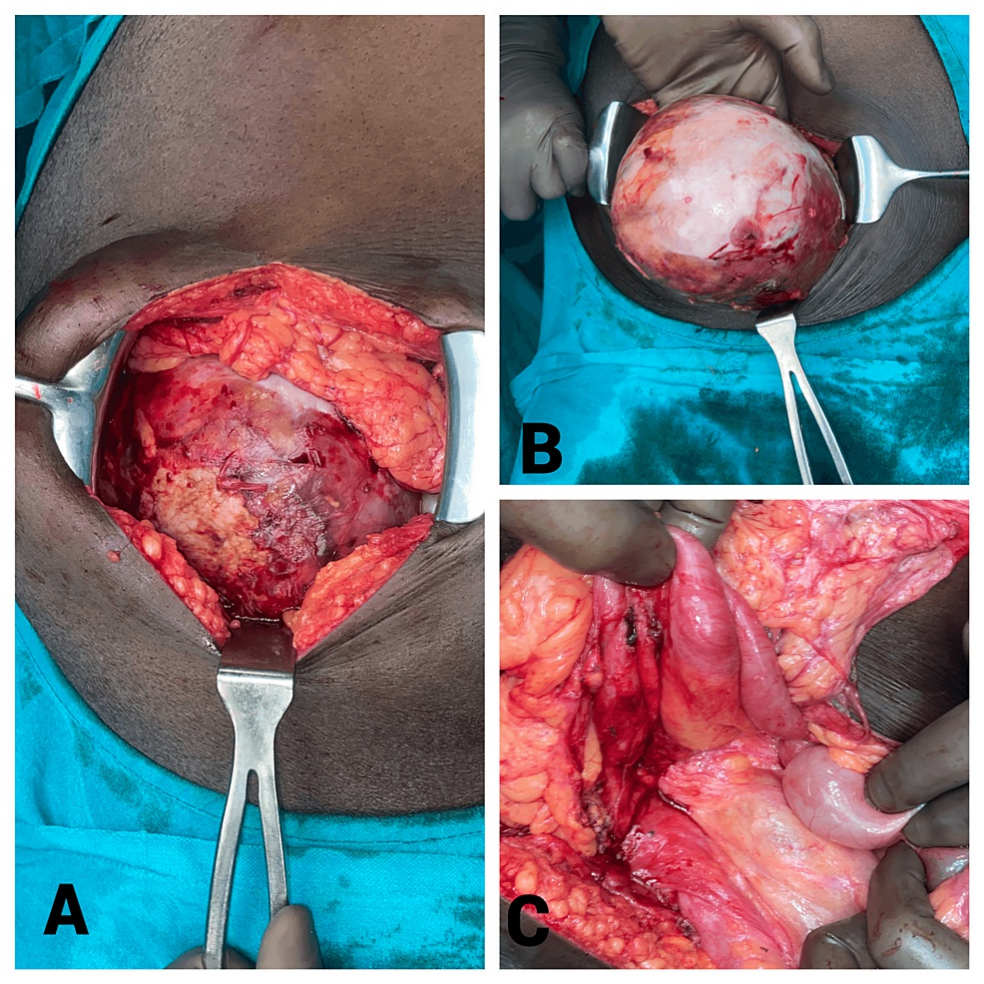
Intra-operative images showing the intact excision of the cyst 3A shows a large (~15x12 cm) cyst on entering the peritoneal cavity. Post-adhesiolysis, the cyst could be moved out of the peritoneal cavity (3B). The rest of the bowel was splayed around it and was healthy (3C).

The patient was given the albendazole regimen for hydatid disease and planned for surgery. He underwent exploratory laparotomy with all due precautions for intra-op spillage and anaphylaxis. An intact excision of the cyst was performed, which was arising from the terminal ileal mesentery with the least disruption of other intra-abdominal contents as shown in Figure 3.

**Figure 4:**
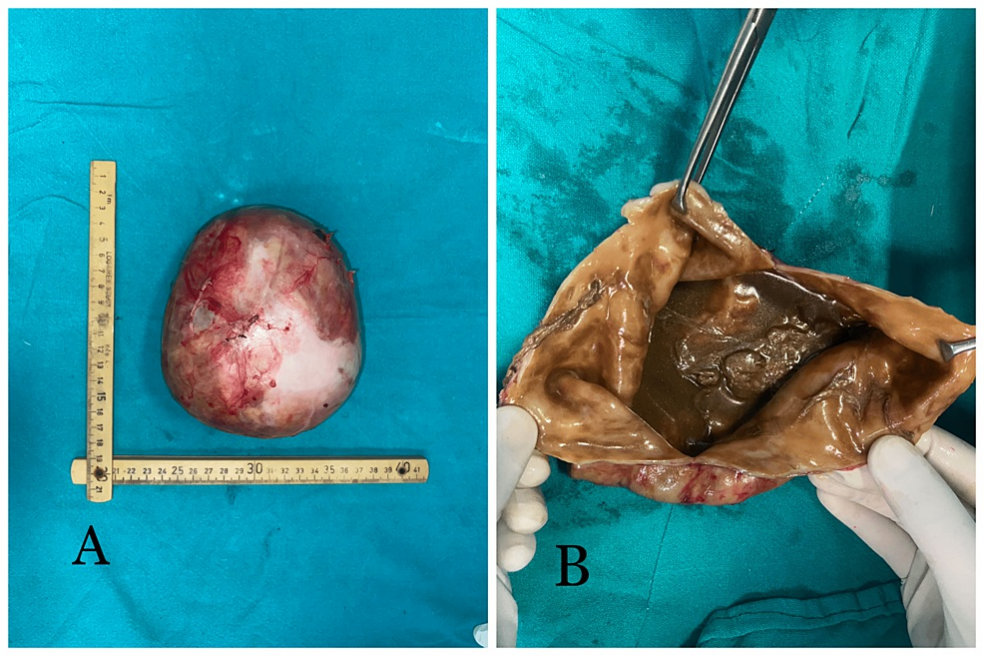
Intact mesenteric cyst as the excised specimen 4A depicts a mesenteric cyst of dimension 15 cm x 12 cm and weighing 2.5 kg. On cutting it open, the mesenteric cyst is found to be containing brown turbid fluid (4B). It is thin-walled with fine rugosity present over the luminal surface.

The specimen measured 15 cm x 12 cm (Figure 4A) and weighed 2.5 kg. On cutting it open, it was filled with grayish-brown turbid fluid. The mesenteric cyst's wall was thin with few rugosities as seen in Figure 4B.

**Figure 5:**
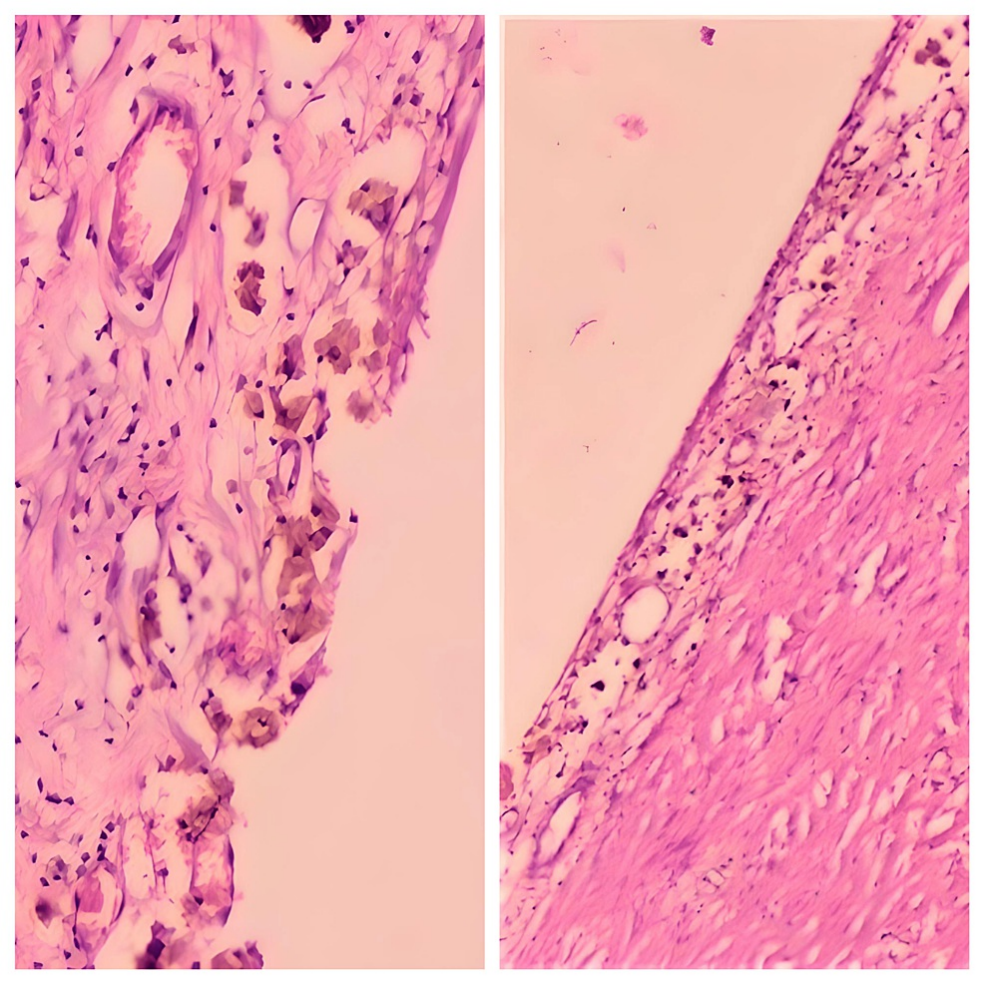
Photomicrographs from the histopathological examination In 5A, the photomicrograph (Haematoxylin & Eosin stain, 100x magnification) shows a collagenous cyst wall infiltrated by a few lymphocytes with the presence of hemosiderin-laden histocytes in the luminal surface of the cyst wall. In 5B (Haematoxylin & Eosin stain, 40x magnification), the photomicrograph shows a collagenous cyst wall without lining epithelium.

The histopathology showed a collagenous cyst wall infiltrated by a few lymphocytes with the presence of hemosiderin-laden histocytes in the luminal surface of the cyst wall (Figure 5). The cyst wall also lacked lining epithelium, and the histopathological impression was a mesenteric pseudocyst. The patient did well postoperatively and did not develop any complications.

## Discussion

A mesenteric cyst is a rare type of abdominal cyst that arises from the mesentery, a fold of the peritoneum that attaches the intestines to the back of the abdominal wall. These cysts can be classified as true cysts or pseudocysts based on their histological features [5]. True mesenteric cysts are lined by epithelium while pseudocysts are not.

Pseudocysts are thought to arise from a variety of etiologies, including trauma, inflammation, and pancreatitis. Pancreatic pseudocysts are well-known complications of acute or chronic pancreatitis, which result from pancreatic duct disruption with the accumulation of pancreatic juice in the peripancreatic space. Over time, the fluid within the pseudocyst can become encapsulated by a fibrous wall, creating a pseudocyst [6].

Mesenteric pseudocysts, on the other hand, are a relatively rare condition, and their etiology is not entirely clear. Some case reports suggest that mesenteric pseudocysts may arise from trauma while others suggest they may be caused by inflammatory or neoplastic processes. A case report by Micković S et al. (2014) described a mesenteric pseudocyst in a patient with a history of abdominal trauma [5].

The diagnosis of mesenteric pseudocysts is challenging, as they may be asymptomatic and reported incidentally on imaging studies. The symptoms, when occur, include abdominal pain, nausea, vomiting, and bowel obstruction [1]. Conditions like lymphoma or GIST may mimic mesenteric cysts. Relevant blood tests and incisional biopsy are needed to rule them out. In some cases, the cyst may become infected, leading to fever and leukocytosis.

The management of mesenteric pseudocysts depends on the size and location of the cyst, as well as the presence or absence of symptoms. Small, asymptomatic cysts may be monitored with periodic imaging studies while larger or symptomatic cysts require surgery. Cyst rupture, torsion, or hemorrhage requires urgent intervention. Complete surgical excision is the gold standard treatment for mesenteric cysts and pseudocysts [7]. The patient’s overall poor health status and the difficult location of the cyst limit us to its drainage.

In our case, preoperatively, we were in a diagnostic dilemma with both a peritoneal hydatid cyst and a mesenteric cyst in consideration in a middle-aged gentleman. Optimizing the safety of our patient, we opted for a laparotomy incision in view of minimizing spillage and achieving intact excision of the cyst. Better mobilization of the cyst and meticulous adhesiolysis helped our objective with the open technique. The postoperative diagnosis was of a mesenteric pseudocyst. Echinococcus IgG could be falsely elevated in cases of past hydatid infection or due to other helminthic infections, which probably happened in this case [8].

However, laparoscopic excision is the preferred surgical approach, as shown by Kurnicki J et al. (2011) and Bhandarwar AH et al. (2013) [9,10]. Bhullar JS et al. (2014) reported an en-bloc resection of the mesenteric pseudocyst with a laparotomy incision preceded by diagnostic laparoscopy [11].

## Conclusions

This case highlights the importance of considering mesenteric pseudocysts in the differential diagnosis of abdominal masses and optimizing the surgical approach on a case-to-case basis. The definitive treatment of mesenteric pseudocysts is surgical excision, which was successfully performed in this case. The prognosis is generally good, with a low risk of recurrence, if the cyst is completely removed.
